# Scientific Items

**Published:** 1880-06-20

**Authors:** 


### SCIENTIFIC ITEMS.

**The Sun's Rays as a Means of Research.**—The Photographic News reports that M. Raoul Pietet is about to try, on an extensive scale experiment, having for its object the disassociation of the metalloids by means of the sun's rays. By the use of a huge metallic mirror, he hopes to bring such a concentration of sun rays to bear upon the metalloids as will enable him to definitely determine the truth or fallacy of the theories of Messrs. Lockyer and Victor Meyer. The experiment will probably be made at Geneva, and will, by reason of its magnitude, be unique for a research of this nature.—*Physician and Patient.*

**Telephonic Transmission through the Human Body.**—It is stated in Chamber's Journal that during some recent experiments in Glasgow it was proved that telephonic sound can be conveyed through a less facile conductor than the usual unbroken wire. In this case a break in the wire was taken up by a small circle of ladies and gentlemen, who joined hands and thus continued the electric current through their own bodies. The effect of interposing these human links was to diffuse and weaken the electric power; but the current was still sufficient to convey some audible reproduction of a song from the transmitting to the receiving end of a telephone.

**The Telephone in Scotland.**—We understand, says the Glasgow Herald, that an experiment upon the longest line of wire yet attempted to be telephonically connected in the British Isles has just been made with the Edison telephones on a private line between No. 7 Sauchiehall Street, Glasgow, and Grangemouth, distant a little over twenty-seven miles. Complete communication was established in about half an hour; and so successful was the operation that messages spoken from Glasgow to Grangemouth, and *vice versa*, were as distinctly audible at the other end as if uttered aloud in an adjoining room. It is added that a similar trial between Glasgow and Edinburgh only awaits the consent of the railway company.

**Cold as a Motor.**—Mr. John Gamgee has halted long enough in Louisville to allow his wonderful genius to develop a marvelous machine for the reduction of temperature in ordinary chambers, and for the ventilation of sick rooms, by the use of cold as a motor. He demonstrates by a beautiful experiment that a small piece of ice will drive a constant current of air through any ordinary room having two openings. He proposes to apply this to the ventilation of rooms of patients sick with contagious diseases, and, by the interposition of such chemical agents as are known to destroy septic matters, force all the vitiated air through such filters as will render it perfectly innocuous, thus bottling the poisonous effluvia until it can be rendered harmless. This is no vagary of the imagination; we have seen the experimental tests so often made that we are sure Mr. Gamgee's will drive air through a room in any desired direction.

**Sanitas.**—Russian turpentine and water are placed in huge earthenware jars, surrounded by hot water. Air is driven through the mixture in the jars continually for three hundred hours, the result being a decomposition of the turpentine, and the formation of a watery solution of the substance, to which Dr. Kingsett, the discoverer, has given the name of "Sanitas." After evaporation, the substance, as sold in tin cans is a light brown powder, of a pleasant taste and odor, and capable in a very remarkable degree of preventing or arresting putrefactive changes. This new disinfectant has been in use for some time in England, and is highly spoken of. It is said to have a pleasant odor, is not poisonous, and does not injure clothing, furniture, etc. For household uses it would seem to be well adapted.–*Scientific American.*

**More Scotch Diamonds.**—Just now the Scotch chemists, especially in Glasgow and vicinity, appear to have "atificial diamonds" on the brain. Mr. Mactear and Mr. Hannay are both Glasgow men; and now Mr. G. C. Stewart, of Greenock, thirty miles down the Clyde, says that he will soon be able to produce "millions of diamonds" of ordinary size every day. The machinery is not yet perfected, but he is going to exhibit his diamonds to the Royal Society "shortly." His is a "very simple chemical process."

**A Transparent Fish.**—A very remarkable fish was captured here on the 21st instant by Mr. O. Blossom. It is about ten feet in length, and its weight is estimated at about four hundred pounds. It is perfectly transparent, and the action of the heart and other functional organs can be plainly seen. Altogether, it is a very remarkable specimen of the finny tribe, and is well worth the attention of scientists and naturalists. Mr. Blossom will arrange a tank containing alcohol in order to preserve it.—*Mackinac, Mich., lettei to Chicago Times.*

**Engineering Inventions.**—Mr. Allen T. Miller, of Philadelphia, Pa., has patented an improved guard for car wheels, the use of which, it is claimed, will render it impossible for any person or anything to be run over by the wheels.

Mr. Strafford C. Hallock, of Yaphank, N. Y., has invented an improved snow plow, which is so constructed as to raise the snow and discharge it at the sides of the track, however solid it may be packed. The invention consists in the combination of three shovels, placed one above the other, with their rear ends farther apart than their forward ends.

Mr. James Robson, of North Shields, County of Northumberland, England, has patented an improved gas engine. The invention consists in employing a piston and rod working in a cylinder. The instroke of the piston is used to draw in on one side of it a charge of gas or vapor and air. On the return stroke this charge is forced through passages into a combustion reservoir, and there retained until the piston returns to the back end of the cylinder. The reservoir is then made to communicate with the back or opposite side of the piston. The gases in the reservoir are then exploded by a flame; their expansion drives the piston forward, which, by its rod and connecting rod to the crank, turns the shaft and fly wheel. On the return of the piston the products of combustion are allowed to escape.—*Scient. Ameri.*

